# ORGANIZATIONAL AND POLICY FACTORS INFLUENCING FEEDING IN RESIDENTS WITH DEMENTIA: NURSING ASSISTANTS’ EXPERIENCES

**DOI:** 10.1093/geroni/igz038.2584

**Published:** 2019-11-08

**Authors:** Joy W Douglas, Seung Eun Jung, Hyunjin Noh, Amy Ellis, Christine Ferguson

**Affiliations:** 1 University of Alabama, Tuscaloosa, Alabama, United States; 2 The University of Alabama School of Social Work, Tuscaloosa, Alabama, United States

## Abstract

In the United States, Certified Nursing Assistants (CNAs) are critical in providing direct care to nursing home residents with dementia, which includes the challenging task of feeding residents. Guided by the Social Ecological Model (SEM), this qualitative study aimed to gain an in-depth understanding of organizational and policy constructs that CNAs encounter when feeding residents with dementia. Using purposive sampling, nine semi-structured focus groups were conducted with 53 CNAs. Each participant had at least one year of experience working as a CNA with older adults. Focus groups were audio recorded and transcribed verbatim. Data were analyzed using the directed content analysis. Factors that emerged were organized into organizational and policy categories within the SEM levels. CNAs identified organizational barriers such as exclusion from the interdisciplinary team, inability to meet resident needs and wants due to budgetary constraints, and inadequate staffing to function efficiently. Organizational facilitators included teamwork, interdisciplinary assistance, and varying dining styles and meal times to accommodate resident needs. Policy-related barriers included funding concerns, staffing ratios, and frustration with unrealistic regulations and state inspections. These results suggest that organizational and policy factors have a large influence on the ease of feeding nursing home residents with dementia. Involving CNAs in interdisciplinary collaboration, resident-centered accommodations, and subtler state inspection behaviors could improve the mealtime experience for both residents and CNAs. Careful attention to these factors may enhance facilitators and minimize barriers to improve the feeding experience of CNAs and residents with dementia.

